# The 2019 and 2021 International Workshops on Alport Syndrome

**DOI:** 10.1038/s41431-022-01075-0

**Published:** 2022-03-09

**Authors:** Sergio Daga, Jie Ding, Constantinos Deltas, Judy Savige, Beata S. Lipska-Ziętkiewicz, Julia Hoefele, Frances Flinter, Daniel P. Gale, Marina Aksenova, Hirofumi Kai, Laura Perin, Moumita Barua, Roser Torra, Jeff H. Miner, Laura Massella, Danica Galešić Ljubanović, Rachel Lennon, Andrè B. Weinstock, Bertrand Knebelmann, Agne Cerkauskaite, Susie Gear, Oliver Gross, A. Neil Turner, Margherita Baldassarri, Anna Maria Pinto, Alessandra Renieri

**Affiliations:** 1 Medical Genetics, University of Siena, Siena, Italy; 2 Med Biotech Hub and Competence Center, Department of Medical Biotechnologies, University of Siena, Siena, Italy; 3 Peking University First Hospital, Beijing, China; 4 Biobank.cy Center of Excellence in Biobanking and Biomedical Research and University of Cyprus Medical School, Nicosia, Cyprus; 5 Department of Medicine, Melbourne and Northern Health, The University of Melbourne, Parkville, VIC 3050 Australia; 6 Rare Diseases Centre, Clinical Genetics Unit, Department of Biology and Medical Genetics, Medical University of Gdańsk, Gdansk, Poland; 7 Institute of Human Genetics, Klinikum rechts der Isar, Technical University of Munich, School of Medicine, Munich, Germany; 8 Department of Clinical Genetics, Guys’ and St Thomas’ NHS Foundation Trust, London, UK; 9 Department of Renal Medicine, University College London, London, UK; 10 Rare Renal Disease Registry, UK Renal Registry, Bristol, UK; 11 Y. Veltischev Research and Clinical Institute for Pediatrics at the Pirogov Russian National Research Medical University, Taldomskaya Street, 2, Moscow, 125412 Russia; 12 Department of Molecular Medicine, Kumamoto University, Kumamoto, Japan; 13 GOFARR Laboratory for Organ Regenerative Research and Cell Therapeutics in Urology, Saban Research Institute, Division of Urology, Children’s Hospital Los Angeles, Los Angeles, CA USA; 14 Department of Urology, Keck School of Medicine, University of Southern California, Los Angeles, CA USA; 15 Toronto General Hospital, Toronto General Research Institute, University of Toronto, Toronto, ON Canada; 16 Inherited Kidney Diseases, Nephrology Department, Fundació Puigvert, IIB-Sant Pau, Medicine Department, Universitat Autónoma de Barcelona, Barcelona, Spain; 17 Division of Nephrology, Washington University School of Medicine, St. Louis, MO 63110 USA; 18 Division of Nephrology, Department of Pediatric Subspecialties, Bambino Gesù Children’s Hospital, IRCCS, Rome, Italy; 19 University of Zagreb School of Medicine, Department of Pathology and Department of Nephropathology and Electron Microscopy Dubrava University Hospital, Zagreb, Croatia; 20 Wellcome Centre for Cell-Matrix Research, Division of Cell-Matrix Biology and Regenerative Medicine, School of Biological Sciences, Faculty of Biology Medicine and Health, The University of Manchester, Manchester Academic Health Science Centre, Manchester, UK; 21 Alport Syndrome Foundation, Phoenix, AZ USA; 22 Nephrology Department, Reference Center for Inherited Kidney Diseases (MARHEA), APHP, Necker Hospital, Paris University, Paris, France; 23 Faculty of Medicine, Vilnius University, Vilnius, Lithuania; 24 Vilnius University Hospital Santaros Klinikos, Vilnius, Lithuania; 25 Alport UK, Tetbury, UK; 26 Department of Nephrology and Rheumatology, University Medicine Goettingen, Gottingen, Germany; 27 Centre for Inflammation, University of Edinburgh, Edinburgh, UK; 28 Genetica Medica, Azienda Ospedaliero-Universitaria Senese, Siena, Italy

**Keywords:** Genetics research, Genetics

## Abstract

In 1927 Arthur Cecil Alport, a South African physician, described a British family with an inherited form of kidney disease that affected males more severely than females and was sometimes associated with hearing loss. In 1961, the eponymous name Alport syndrome was adopted. In the late twentieth century three genes responsible for the disease were discovered: *COL4A3*, *COL4A4*, and *COL4A5* encoding for the α3, α4, α5 polypeptide chains of type IV collagen, respectively. These chains assemble to form heterotrimers of type IV collagen in the glomerular basement membrane. Scientists, clinicians, patient representatives and their families, and pharma companies attended the 2019 International Workshop on Alport Syndrome, held in Siena, Italy, from October 22 to 26, and the 2021 online Workshop from November 30 to December 4. The main topics included: disease re-naming, acknowledging the need to identify an appropriate term able to reflect considerable clinical variability; a strategy for increasing the molecular diagnostic rate; genotype-phenotype correlation from monogenic to digenic forms; new therapeutics and new therapeutic approaches; and gene therapy using gene editing. The exceptional collaborative climate that was established in the magical medieval setting of Siena continued in the online workshop of 2021. Conditions were established for collaborations between leading experts in the sector, including patients and drug companies, with the aim of identifying a cure for Alport syndrome.

## Introduction

Alport syndrome (AS) is a clinically heterogeneous nephropathy caused by pathogenic variants in collagen IV genes (*COL4A3*, *COL4A4*, *COL4A5*) with a prevalence of 1:5.000 [1]. The collagen α3α4α5(IV) heterotrimer represents an essential constituent of the mature glomerular basement membrane (GBM) and is only synthesised and secreted by podocytes in the kidney [2]. GBM disruption leads to microscopic haematuria, proteinuria and progressive fibrosis, advancing to end stage kidney disease (ESKD) in many cases. Extra-renal manifestations include cochlear (bilateral high-frequency sensorineural hearing loss) and ocular abnormalities (anterior lenticonus and perimacular flecks), which often contribute to the clinical phenotype. Established therapies include the use of renin–angiotensin–aldosterone inhibitors (RAAS) as standard of care, which delay the progression to ESKD. For progressive forms that result in ESKD, the only options are dialysis and kidney transplantation, the latter being restricted by the availability of compatible donors, access inequity in developing nations and by the necessity for life-long immunosuppressive treatment.

The 2019 International Workshop on AS was held at the University of Siena, Italy, from October 22–24, while the 2021 Workshop was held online due to COVID-19 pandemic.

These meetings were organised through the concerted efforts of patient advocacy groups plus physicians, scientists, and researchers from around the world. The Workshops focused on the latest findings in the field of basic science, the importance of considering the involvement of other organs such as eyes and ears in AS pathology, and on new therapeutic perspectives. The most powerful aspect of these Workshops was their unique opportunity to provide a collaborative forum for participants from a wide range of backgrounds from around the world, thus highlighting progress made since another two Workshops, 30 years ago [3]. In Siena, this forum enabled a large group of AS patients representing different ages, genotypes, cultures, and experiences to request that their voices be received more openly by the experts who study and treat AS. Specifically, they expressed their hope for a patient-centric shift in the way that AS is diagnosed, treated, and understood. They also raised the issue of classifying AS as a spectrum disorder. The following topics were covered throughout the two Workshops.

## Genotype-phenotype correlation from monogenic to digenic forms

The first day of this well-established biennial Workshop on AS is always dedicated to genetics. In the last two meetings this was under the leadership of CD, FF, JH, BSLZ, JS and AR. The term AS (ORPHA:63) encompasses a group of inherited kidney diseases characterised by haematuria and proteinuria, progressive kidney failure, hearing impairment and ocular abnormalities. Inheritance is X-linked (due to a disease-causing *COL4A5* variants, (OMIM#301050, ORPHA:88917), or Autosomal Recessive (AR) (biallelic disease-causing variants in the COL4A3 or *COL4A4* gene; OMIM#203780; ORPHA:88918) [4]. In recent years, the presence of compound heterozygous (sometimes referred to as digenic) inheritance has been reported, with disease-causing variants in two of the three *COL4A3–5* genes [5–12]. Modifying variants affecting other podocyte or GBM genes can worsen the phenotype [13].

Autosomal Dominant (AD) inheritance (with a single disease-causing variant in *COL4A3* or *COL4A4* OMIM#104200; ORPHA:88919) is also reported, but some prefer to avoid the term Alport ‘syndrome’ for this last group. There were constructive discussions about a revised nomenclature that indicates individuals with a heterozygous disease-causing variant do not have a syndromic form of the disease, since they are much less likely to develop kidney failure, and do not usually develop hearing loss or ocular abnormalities [12]. Recent experience suggests that disease-causing *COL4A3–5* variants are associated with a broad phenotypic spectrum, which is also influenced by nephroprotective therapy [14]. These include persistent microscopic haematuria with a similar family history of microscopic haematuria, but also focal segmental glomerulosclerosis (FSGS), and kidney failure [15], familial IgA glomerulonephritis [16] and possibly other types of immune-mediated glomerulonephritis, as well as cystic kidney disease [17, 18] and kidney dysplasia [19]. However, in all these conditions, persistent microscopic haematuria and a family history or microscopic haematuria or kidney failure may be present, suggesting an underlying disease-causing *COL4A3–5* variant.

Analysis of *COL4A3–5* pathogenic variants in the gnomAD database has revealed that X-linked AS occurs in about one in 2000 individuals, and single heterozygous *COL4A3* or *COL4A4* variants in about one in 100 [20]. XL and AR AS are still rare diseases, but the “Alport spectrum” including the XL, AR, AD and double heterozygous (digenic) forms is now the commonest cause of inherited kidney disease, and the second commonest cause of inherited kidney failure.

Interestingly, a recent update of the LOVD database has demonstrated that the mean age at kidney failure for males with X-linked disease is now 29 years, whereas it was 25 years in 2016. This might be because of the beneficial effects of widespread use of RAAS inhibitors, or as a consequence that the disease-causing variants detected recently are milder (because families with early onset kidney failure and more “severe” variants have already been diagnosed).

Three types of more recently identified disease-causing *COL4A3–5* variants were discussed in detail: hypomorphic, digenic and modifying variants.

Hypomorphic disease-causing variants result in a milder phenotype, but there is no formal definition. Thus, a hypomorphic disease-causing *COL4A5* variant may result in kidney failure in late middle age in a man, and a hypomorphic heterozygous disease-causing *COL4A3* or *COL4A4* variant may not be associated with microscopic haematuria, and/or lead to symptomatic disease only if found in *trans* with a second, overt, disease-causing variant. Typical hypomorphic disease-causing variants include many missense changes, for example some position 1 Gly substitutions with Ala, Ser or Cys, as well as non-Gly substitutions [21]. In addition, Gly substitutions adjacent to the non-collagenous domains or non-collagenous interruptions also result in a milder phenotype. Two common less severe disease-causing variants are Gly624Asp in *COL4A5* (which is associated with late onset kidney failure) [22] and Leu1474Pro in *COL4A3* (which may not on its own cause microscopic haematuria, but can be associated with kidney failure when found together with a second disease-causing *COL4A3* variant in *trans*, or due to digenic inheritance).

Despite the Gly624Asp variant being estimated less severe, recent evidence has highlighted cases in which the severity of the variant is associated with clear evidence of kidney insufficiency and/or ESRD.

Macheroux et al. [23] recently report how this variant is the genetic cause that reported for an unexpected severe phenotype in a X-linked family where the female proband shows microscopic haematuria and proteinuria at the age of 20 and 41 years, respectively. The microscopic haematuria was also present in the daughter (from 6th month of life), the son (from 22nd month of life), the mother and the maternal grandniece, while the proteinuria was detected in the maternal aunt and paternal grandmother.

Boeckhaus et al. [24] in a prospective cohort study underline how hemizygous patients for this variant have an increased risk developing ESRD if they do not receive pharmacological treatment, while in patients treated early a reduced risk of reaching the endpoint is reported thanks to the nephroprotective action of ACEis.

Furthermore, in the EARLY PRO-TECT Alport trial it is reported that two of eight children with the variant included in the study showed progress of the disease during the trial [25].

All these reports and studies illustrate the difficulties faced when interpreting the clinical significance of the variant which should be considered a milder form of the disease, but still causing kidney failure in midlife.

Double heterozygous (digenic) variants are variants in the *COL4A3* and COL4A4 gene. This observation is very rare and has generally only been detected since exome sequencing has allowed the examination of multiple genes simultaneously. The inheritance of *COL4A3* and *COL4A4* double heterozygous variants is complex, because the variants may be inherited on the same chromosome (in *cis*) where inheritance is AD, or on different chromosomes (in *trans*) where inheritance is AR [5, 6]. The renal outcome appears to be worse with a combination of heterozygous *COL4A3* and *COL4A4* variants than with a single variant; however, further studies are needed [10]. Recent advice is that these individuals should also be treated with RAAS blockade if they have albuminuria or additional risk factors for progressive kidney disease such as hypertension [26], and other first-degree family members should be offered cascade genetics testing. The suitability of individuals with heterozygous variants to act as kidney donors continues to be contentious: they are at increased risk of renal disease, but those who are older, normotensive and without microalbuminuria may be highly motivated to give a kidney to a relative, particularly when there are no suitable alternatives.

The co-inheritance of additional genetic modifiers that only exert their negative effect on the background of a primary collagen IV variant may explain the adverse outcome seen in some patients [27–29]. The term “modifying” variants describes the association of a disease-causing variant in one of the *COL4A3–5* genes, with a coincidental variant in a GBM or podocyte gene [30]. Such heterozygous variants in many GBM or podocyte genes are not usually disease-causing on their own, either because they are recessive, or they are hypomorphic and this continues to be an area of active research.

## Disease re-naming, with the search for an appropriate term that encompasses the large clinical variability

There is an international initiative to adopt a more consistent approach to naming genetic diseases: https://clinicalgenome.org. Agreement on not using the term “carriers” for *COL4A5* heterozygous females has been reached, and there is a growing consensus on avoiding the same term for *COL4A3/A4* heterozygotes. This is because there is a real risk of clinically significant renal impairment in heterozygous subjects [31] who should be made aware of the necessity for regular clinical check-ups. JS presented data from large-scale population sequencing initiatives, indicating that the prevalence of rare heterozygous *COL4A3/4/5* variants that are predicted to be disease-causing, and are strongly associated with the presence of persistent haematuria, is as high as 0.94% in the UK population [20].

This is a very high percentage and, at the same time, it implies that the risk of EKD is very low in individuals not ascertained by the presence of a personal or family history of kidney disease who harbour such variants. Many of these individuals may only have isolated microscopic haematuria for their entire life, or even be completely asymptomatic. Therefore, it is necessary that the status of heterozygous subjects (especially in the absence of features such as proteinuria, chronic kidney disease or hypertension that suggest underlying kidney damage) is not perceived as a devastating event. The term “Alport Syndrome” as a diagnostic label for every patient with a COL4 variant is unhelpful, as it fails to distinguish between those at high risk of renal disease with other characteristic extra-renal features, and those who are at very low risk of any significant problems. Alicia Byrne facilitated an international discussion and the alternative name “Alport Spectrum” was preferred. If *COL4A3/COL4A4* heterozygous individuals, who are so commonly identifiable in the population, are included under the umbrella description of Alport spectrum, the condition would no longer be regarded as rare. Some patients with syndromic disease (including the extra-renal manifestations) may still prefer the label AS. These proposals are now under consideration by the wider community.

## What to do when Alport syndrome is strongly suspected, but a pathogenic variant is not identified

Four approaches are commonly used to detect *COL4A3–5* variants: exome sequencing, panel diagnostics, Multiple Ligation-dependent Probe Amplification and Sanger sequencing. In 2021, a consensus statement refining the ACMG criteria on standards and guidelines for the molecular diagnostics of AS was published [32].

The detection rate of disease-causing variants is higher in individuals with suspected X-linked AS (up to 95%) than in those with a suspected heterozygous disease-causing *COL4A3* or *COL4A4* variant (where it may be only 50%). This is probably because the clinical features of X-linked disease are more distinctive with microscopic haematuria, kidney failure, and, often, hearing loss and ocular abnormalities, in addition to a suggestive family history. On the other hand, disease-causing variants may be found less frequently in *COL4A3* and *COL4A4*, because these cohorts include individuals with possibly other causes of persistent microscopic haematuria, including IgA, post-streptococcal and C3 nephropathy.

Other explanations for a reduced detection rate include reduced coverage of collagen IV exons (especially exons 5 and 6) and the fact that large deletions (>50 bp) may be missed with exome sequencing in some laboratories. Although the critical nature of position 1 Gly residues is now recognised, the significance of many variants affecting the carboxy non-collagenous domain is still unknown.

There was no consensus on the strategy to address the “missing variants”. There are in silico methods to detect large deletions if these are not detected with exome sequencing, and there is now an in vitro method for confirming collagen IV variant pathogenicity, but it is still a research tool. It is important to keep in mind synonymous variants (which may affect a splice site), other non-canonical splice variants, and less severe variants such as p.Leu1474Pro in *COL4A3*. Some variants are difficult to call conclusively as disease-causing, and Weisen Xia presented her research on using AI to interpret pathogenicity. It is possible to check for splicing variants using a minigene assay and urinary podocytes or skin or hair roots, but splicing may be tissue specific. Moreover, the limitation in variant detection can be explained by incomplete coverage of the studied region, the presence of a deep intronic variant [33], variants of uncertain significance, Copy Number Variation or other types of structural variants that may not be detectable using panel, exome or other targeted sequencing assays.

Most laboratories represented reported that pathogenic variants in other FSGS genes were uncommon when AS is suspected clinically [34]. When a diagnosis of AS is strongly suspected clinically, but no variant is detected following DNA analysis, clinical review may be helpful, including re-examination of a kidney biopsy to look for lamellation in the proband or an affected family member, retinal imaging and optical coherence tomography. Finally, genetic linkage studies can exclude a gene if DNA is available from suitable relatives whose phenotype is known.

If not already performed, a kidney biopsy may be necessary if the genetic cause of kidney disease cannot be established by DNA analysis, and/or there is an atypical clinical course in a patient with AS, in order to exclude other glomerular and tubulointerstitial kidney diseases such as IgA nephropathy (which has strong genetic risk factors and can occur in familial clusters) [35], membranous nephropathy and interstitial nephritis. Even if electron microscopy is mandatory to confirm the diagnosis of AS, light microscopy plays an important role in studying the interstitium, whose involvement could be predictive of more severe progression, and also to reveal focal and segmental glomerulosclerosis.

The results of kidney biopsy may strongly support a clinical diagnosis of AS, but do not have the advantage of identifying the underlying disease-causing variant, which is why clinicians increasingly request genetic testing first when considering a diagnosis of AS.

The presence of a glomerular disease (primary membranoproliferative GN/C3 glomerulopathy, IgA nephropathy) does not exclude AS [16, 36, 37]. The association between AS and immune glomerular diseases suggest that the abnormal GBM resulting from the presence of pathogenic *COL4A3/A4/A5* variants may predispose to immune-mediated injury. Recently JS and Philip Harrak reported that how often both diseases occur together is unknown because few individuals with IgA glomerulonephritis routinely undergo electron microscopy for GBM thinning or testing for genetic variants. On the other hand, heterozygous *COL4A3* and *COL4A4* variants and IgA disease each affect ~1% of the population, so the conditions might coexist by chance in 0.01% [38]. Kohei Omachi (Washington University, St. Louis, USA), from the group of Jeff H. Miner, reported on ways to detect and assess the pathogenicity of novel variants, having established a collagen alpha 345(IV) heterotrimer formation assay that is amenable to high throughput screening by using a split NanoLuc luciferase complementarity system [39]. The assay provided ways to determine the functionality of Gly394Ser, Gly454Ala and Gly960Arg variants in *COL4A4*. The level of luciferase activity revealed the effect that those variants have on impairing NC1 and 7S domain assembly and stability, allowing prediction of the resulting impact on collagen IV network and basement membrane formation. This assay is suitable for studying variants that affect the stability of the heterotrimer, to evaluate the pathogenicity of novel variants, and to find potential candidate compound to correct the mutant α5(IV)-dependent defect of alpha α345 (IV) trimer secretion.

Another strategy for confirming the pathogenicity of new *COL4A5* variants is the creation of animal or podocytes models using CRISPR/Cas9 gene editing, followed by clinical evaluation or measurement of *COL4A5* protein expression by western blot analyses and immunofluorescence assays [40].

## Update on clinical trials

Recent and current clinical trial in AS were reviewed including: (1) the phase 3 randomised, placebo-controlled EARLY PRO-TECT Alport trial; (2) the phase 2 open-label, basket study AFFINITY; (3) the phase 2 randomised, placebo-controlled HERA trial; (4) the phase 2/3 randomised, placebo-controlled CARDINAL trial; and major clinical trials in patients with CKD (but not specific for AS), such as DAPA-CKD and FIDELIO-DKD.

**Fig. 1:**
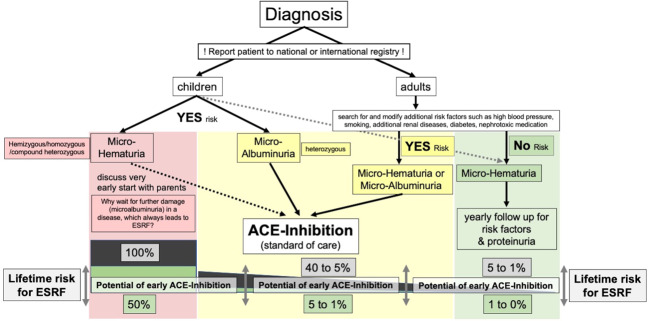
Visual summary of current standard of care treatment recommendations and individual lifetime risk for ESRF according to the gene variant and additional risk factors. Red: patients with—classical‖ Alport syndrome and a 100% risk for early ESRF. Therapy should be initiated at diagnosis (in children 2 years and older) at very early stages of disease. Nephroprotective therapy has the potential to reduce lifetime risk for ESRF to 50% in those with less severe missense variants. Yellow: heterozygous patients with an intermediate lifetime risk of 5 to 40% for ESRF. If therapy is initiated early at microalbuminuria (or even micro-haematuria in patients with additional risk factors), nephroprotective therapy has the potential to reduce lifetime risk to 1 to 5%, only. Green: heterozygous individuals with a low risk for ESRF, which is <1%, if patients remain in a disease management programme to check for additional risk factors for their lifetime.

**Fig. 2:**
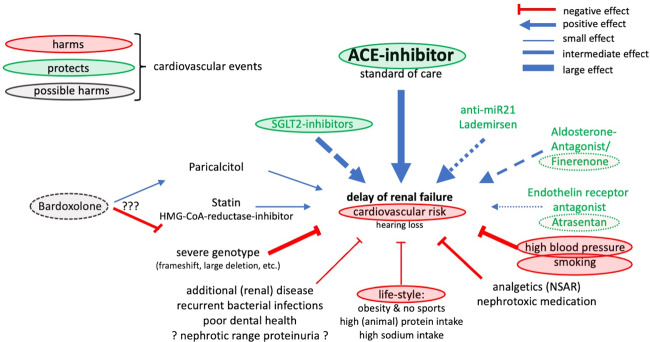
Current and possible future treatment options. Summary of current and possible future treatment options and postulated effects on (the high) cardiovascular risk of patients with Alport syndrome, which also needs to be addressed.

In summary, the EARLY PRO-TECT Alport (NCT01485978) trial in children 2 years and older, at very early stages of AS, demonstrated the safety of Ramipril and revealed evidence of efficacy (i.e. delaying progression of albuminuria) [41]. These findings have led to an update of treatment recommendations for patients with AS [23] (Figs. 1 and 2).

Figure 2 further explains current and future treatment options. In detail, Paricalcitol and HMG-CoA-reductase-inhibitors (Statins) both showed pleiotropic nephroprotective effects in *COL4A3*^−/−^ mice as animal models for AS [42, 43]. Both compounds are part of the treatment recommendations, if indicated because of secondary hyperparathyroidism (Paricalcitol) or elevated cholesterol levels (Statin) [44, 45]. Green circles indicate compounds, which positively influence both, the chronic kidney disease and the high cardiovascular risk in AS. Red circles indicate factors, which negatively influence both, the chronic kidney disease and the high cardiovascular risk in AS. Again, for this reason, a healthy lifestyle (doing sports, moderation of dietary intake of meat protein and salt, and maintaining a body mass index less than 25 kg/m^2^), strict blood pressure control and no smoking are part of the current treatment recommendations [26, 44]. Green letters indicate potential new therapies being evaluated in ongoing clinical phase 2 and 3 trials. At present (February 2022), analysis of the risk-benefit ratio of Bardoxolone Methyl in patients with AS possibly could indicate possible harm.

The AFFINITY study (NCT04573920) with the endothelin receptor antagonist atrasentan in patients with proteinuric glomerular diseases includes 20 patients with AS and an intermediate risk for fast progression. The trial is recruiting currently. Preclinical data in Alport mouse models look promising; however, results of the phase 2 clinical trial are not expected before the end of 2023.

The HERA study (NCT02855268) with the anti-microRNA21 compound lademirsen is specific for AS and includes 45 patients at high risk of fast progression. The trial is recruiting currently. Preclinical data in Alport mouse models look promising, target engagement has been shown in humans; however, results of the phase 2 clinical trial are not expected before the end of 2023.

The CARDINAL study (NCT03019185) with bardoxolone methyl is specific for AS and includes 30 patients in the open-label phase 2 and about 160 in the blinded, placebo-controlled phase 3. Patients in phase 2 study have finished the 2 year (104 weeks) in early 2019, phase 3 have completed week 104 in summer 2020. According to the baseline data, phase 3 includes many patients with a low to intermediate risk for fast progression [46] Bardoxolone has never been tested in Alport mouse models; but there are preclinical data in other CKD mouse models, in which a bardoxolone analogue has caused podocyte damage and increased albuminuria [47]. Results of the phase 2 and phase 3 clinical trial have not yet been published. In a recent meeting of the FDA Cardiovascular and Renal Drugs Advisory Committee, the FDA review team did not assess the phase 3 trial results as positive.

As first in class, the Sodium-Glucose co-Transporter-2 (SGLT2) inhibitor Dapagliflozin has been approved for CKD, because of its profound nephroprotective effect and the impact on cardiovascular risk [48]. EMPA-REG and the ongoing EMPA-KIDNEY study point to a general nephroprotective effect in CKD [49]. However, the very limited number of 6 patients with AS out of more than 4000 other patients in DAPA-CKD means that it is not clear yet whether SGLT2i are specifically beneficial in glomerular diseases such as AS. The panel agreed that SGLT2i seem to be promising add-on therapies for AS patients with a risk of progression, and that they should be prescribed in addition to ACE inhibitors or ARBs where regional regulations permit. In this context, Jan Boeckhaus presented the results of an observational case series in 6 patients with (hereditary) FSGS or AS [50]. Therapy was well tolerated and reduced albuminuria. Interventional trials specific for young patients with AS (including children 10 years and older) at early stages of disease are in preparation. Meanwhile, it is desirable that long term outcomes in treated patients should be monitored (OG at gross.oliver@med.uni-goettingen.de can offer suggestions how). Although preliminary, and not targeted specifically to AS, these results identify SGLT2-i as a very promising new and interesting therapeutic option, which requires further evaluation. Starting in early 2022, the mineral-corticoid receptor antagonist finerenone will be evaluated in FIONA, an international paediatric trial in children with CKD, including AS (Press release: https://www.media.bayer.com/baynews/baynews.nsf/id/Bayer-extends-clinical-development-program-finerenone-Phase-III-study-children-adolescents-chronic?OpenDocument&sessionID=1639500433). Finerenone has been approved for diabetic CKD and is postulated to work in an additive fashion with SGLT2i [51]. Preclinical data in the Alport mouse model have shown an aldosterone escape in Alport mice treated with ramipril, leading to progressive albuminuria and scarring, which might be targeted by mineral-corticoid receptor antagonists [52].

Shota Kaseda (Kumamoto University), from the group of Hirofumi Kai, presented the use of UBE-1099, a reversible Keap-1 inhibitor that inhibits the Keap1-Nrf2 binding inducing downstream Nrf2 activation [53], and evaluated the effect of UBE-1099 in a mouse model of AS. According to transcriptome analysis the UBE-1099 mechanism of improvement seems to be related to the expression of a set of genes associated with cell cycle and cytoskeleton.

## Therapeutic approach using chaperones

Christoforos Odiatis, from the group of Constantinos Deltas, University of Cyprus, presented data supporting the hypothesis that the administration of pharmacological repurposed chaperones may improve clinical progression. This work was done in mice recapitulating AR AS, with the Gly1332Glu *COL4A3* mutation, which is a founder genetic variant amongst Cypriots.

## Gene therapy approaches using gene editing

Gene therapy is now universally recognised as a possible therapeutic application for rare diseases, including AS.

**Fig. 3:**
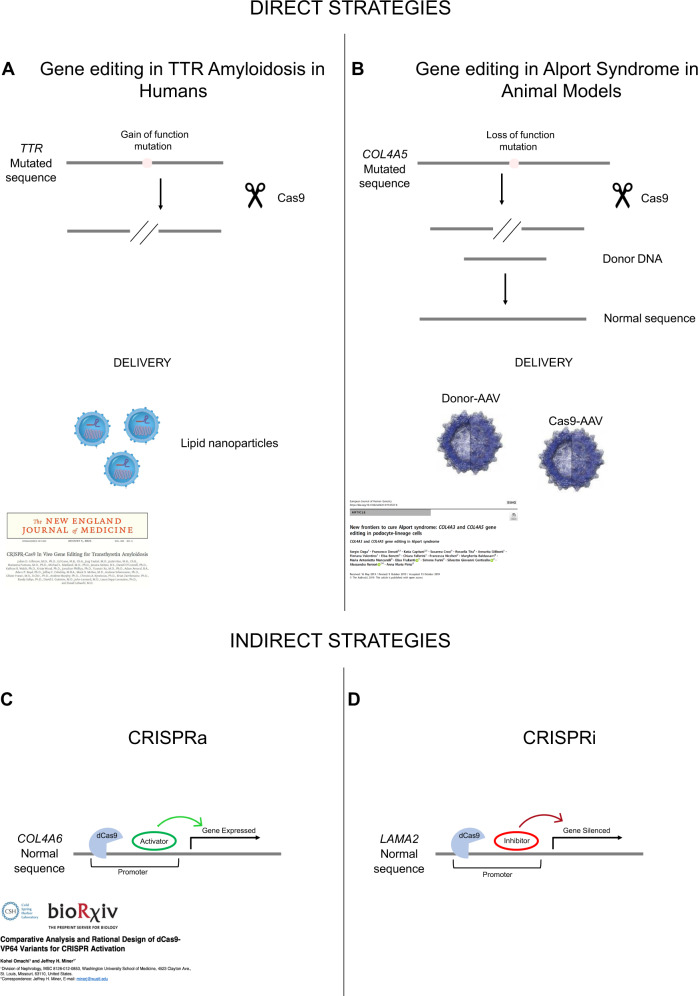
Gene editing approaches: comparison between direct and indirect strategies. The CRISPR/Cas9 direct approaches are targeting straightforward mutated genes (**A**, **B**). The CRISPR/Cas9 indirect approaches are based on the action of endonuclease deficient activity dCas9 fused to either transcriptional activators or repressors that either upregulate (CRISPRa) or silence (CRISPRi) genes (respectively) relevant to the pathophysiology of AS (**C**, **D**). **A** TTR gene mutated sequence is edited in humans by spCas9 alone. Indel (insertion/deletion) is created after the cut and the gain of function variant (the protein precipitates in aggregates dangerous for the cell) is edited in a loss of function allele. **B** The loss of function variant in *COL4A5* gene is edited to the normal sequence, by means of spCas9, in cultured cells and animal models employing CRISPR/Cas9 approach. **C** The *COL4A6* gene, a minor basement membrane gene, is forced by dCas9/CRISPRa to be overexpressed in podocytes, which should replace the missing α3α4α5(IV) network with the α5α5α6(IV) network; this could slow kidney disease progression. **D** The *LAMA2* downstream gene is silenced by the dCas9/CRISPRi in glomerular cells, which should reduce laminin α2 in the GBM and slow kidney disease progression.

Julian Gillmore (Centre for Amyloidosis and Acute Phase Proteins, University College London, UK) presented recently published work exploring a gene editing approach in humans to treat *TTR*-related amyloidosis disease, due to gain-of-function variants (Fig. 3A) [54]. Studies in vitro have proven the ability of the system to match precisely the mutated locus where to direct the Cas9 ribonucleoprotein with low off-target events. Clinical in vivo studies in a small group of patients have shown a durable knockout of *TTR* after the administration of a single dose. Lipid nanoparticles were used as vehicles of the Cas9 system in order to guarantee a very rapid distribution to the liver through the hepatic artery. Furthemore, NTL-2001 was associated with only mild adverse events.

Sergio Daga (Medical Genetics, University of Siena, Siena, Italy), from the group of Aelssandra Renieri, has employed a two-plasmid CRISPR/Cas9 genome editing approach, achieving a beneficial and stable correction of the *COL4* loss-of-function variants on urine-derived podocyte-lineage cells (Fig. 3B) [55, 56]. In order to translate the in-vitro application into a future in-vivo clinical trial, dogs and mice have been used as preclinical animal models to investigate the safety and efficacy of the system. Adeno-Associated-Viruses (AAVs), namely AAV9, are proposed as a delivery system to carry both plasmids of the CRISPR/Cas9 system into the affected kidney of the animals.

Jeff H. Miner (Washington University, St. Louis, USA) described two new promising approaches, namely pro-repair gene activation (CRISPRa) to achieve gene gain of function, and pathogenic gene deactivation or inhibition (CRISPRi) to achieve gene loss of function. These approaches differ from the classical CRISPR/Cas9 approach to directly repair the causative genetic variant of AS. Neither of the two approaches necessitates a DNA cleavage. CRISPRa (Fig. 3C) uses a catalytically dead mutant form of Cas9 (dCas9), whose endonuclease activity is removed through point mutations, fused with transcriptional activator domain to modulate target gene expression. A target gene selective gRNA brings the CRISPRa complex to the genomic locus, and rather than cutting the DNA, the dCas9 complex activates the downstream gene expression [57]. The rationale for the application of the CRISPRa is based on the hypothesis that in AR AS, *COL4A5* and *COL4A6* genes are intact. Employing CRISPRa to activate *COL4A6* gene expression in podocytes would lead to a forced expression followed by production and secretion of α5α5α6(IV) by podocytes, which will normalise the GBM’s structure and function, compared with an altered or absent α3α4α5(IV) heterotrimer. In the CRISPRi approach (Fig. 3D) the dCas9 is fused with a transcriptional repressor such that when a gRNA brings the CRISPRi complex to the genomic locus, it represses the downstream gene expression instead of activating it. Since in Alport GBM, laminin subunit α2 deposition is injurious to podocytes physiology and promotes glomerular disease progression, inactivation of the *LAMA2* gene, mediated by CRISPRi, in glomerular cells could slow disease progression.

**Fig. 4:**
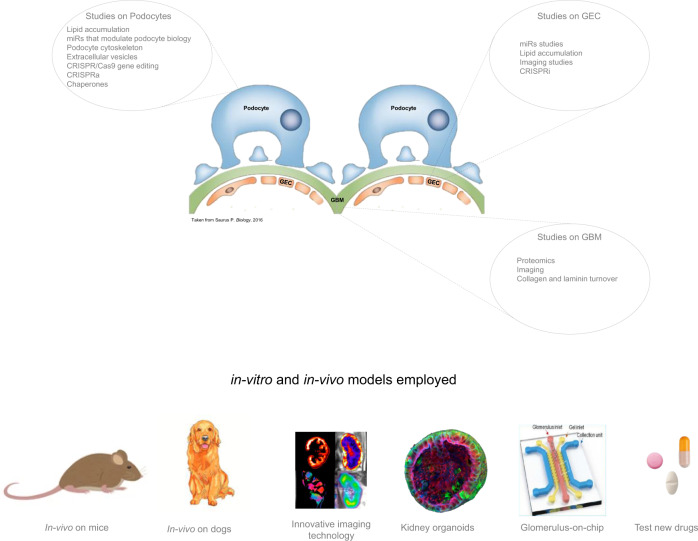
Basic science approaches. In the upper panel are summarised the new studies and approaches on podocytes, GEC and GBM ranging from CRISPR/Cas9 application to miRs that stimulate podocyte biology, proteomics and chaperones in combination with studies on podocytes cytoskeleton. In the lower panel the downstream applications of these: imagine technology, organoids in 3D culture, glomerulus on a chip to mimic the glomerular structure in vitro, and preclinical animal model testing in dog and mice.

Moreover, multiple studies and multiple approaches were presented during these Workshops, identifying new research possibilities on podocytes, on renal glomerular endothelial cells and on GBM, looking for a possible treatments targeted at all the glomerular components involved in AS. The studies proposed range from diversified and innovative in vitro applications, the study of organoid models, and preclinical approaches on mouse and dog models, which are necessary before drugs are approved for clinical trials. New possible drugs need to be also considered in addition to the already widely used (ACE-i or ARBs). All the projects are summarised in a schematic representation in Fig. 4.

## The voice of patient advocacy group

The most powerful aspect of these Workshops is their unique opportunity to provide a collaborative forum for AS patients, caregivers, clinicians, and researchers from around the world. In Siena, this forum enabled a large population of AS patients representing diverse ages, genotypes, cultures, and experiences to request that their voices are more openly received by the experts who study and treat AS. The same spirit was then held online during the second Workshop. Specifically, they hope for a patient-centric shift in how AS is diagnosed, treated, and understood. They also raised the potential of classifying AS as a spectrum disorder.

Although advances in research and treatments to prevent the loss of kidney function obviously remain the most important desire of the AS patient and caregiver community [58], research efforts tend to overlook the unique psychological, sociological, and interpersonal needs of individuals living with AS.

In the last Voice of the Patient meeting, published online by the U.S. Food and Drug Administration (FDA) [58], it is reported that “seventy percent of participants indicated that AS symptoms interfered with their daily life at least moderately”. Their reported experience is indicative of how the majority of Alport patients feel, and how much AS has a psycho-sociological impact on their lives.

For example, they invited conference attendees to consider the negative consequences of implicit biases associated with physiological deafness, as well as the developmental, social, and logistical complications faced by an AS patient who may appear able-bodied but nonetheless cannot adequately perceive human speech. They asked that researchers and clinicians consider being more sensitive in their choice of words while interacting with AS patients who have hearing loss/deafness, and suggested that clinicians might offer assistive aids such as amplification in their medical offices, and particularly at medical events that feature AS-specific topics. Such aids could include providing subtitles, real-time text translation, and sign language interpretation. They also expressed families‘ need for help in identifying possible sources of financial and emotional support when faced with a new diagnosis of deafness/hearing loss. Families reiterated the importance of psychological support for patients and families during the second Workshop. In particular, the representative of the Italian Association (A.S.A.L.) described their proposals aimed at promoting health, understood as a complex system of physical, psychological and social well-being of each individual, such as the Psychological Listening Desk and the Psychological support groups. A high point of the in-person Workshop held in Siena occurred when the father of a child newly diagnosed with AS pointed across the room at patient advocate Sam Clarke and emotionally professed that “Sam was the answer!” to the question of how he could raise his young son to believe he could lead a full and productive life. This father’s fear for his son’s psychological well-being was echoed in testimonies of several patients who described overcoming struggles with isolation after connecting with others who shared similar experiences. During the 2021 online Workshop, the importance of a greater involvement of young patients in these workshops was discussed in depth in order to share experiences and feelings and address the disease together. The representative of the American Association (Alport Syndrome Foundation) presented their colouring book that tells the story of a young girl with AS and her family. It was created as a gift and educational tool for families to encourage conversations with their son/daughter about living with AS.

Anonymised data from the US Alport Syndrome Foundation’s social media website indicate that more than 2100 individuals from more than 70 countries use the site to collaborate, provide peer support, and interact with experts in the community. Associazione Sindrome di Alport facebook page, moderated by the Italian national patient organistion ASAL Onlus, reported over 2300 followers. The China patient organisation reported 1567 patients from 33 provinces in China are connected through WeChat, China’s social media platform. Alport UK reported how Alport Warriors, their closed Facebook page, doubled in size as the community went online during the pandemic and has nearly 900 members from more than 40 countries. The importance of the international Alport Syndrome Alliance was underlined as a global network to advance treatments and knowledge. Currently operated by Alport UK, the alliance will be managed by all the national associations of Alport, representing their national members. Alport UK, organisers of the workshops, and ASAL Onlus will collaborate on a joint project with other Alport national patient organisations to create a resource base for the Alport community. This new project will accelerate collaboration across the community of patients, clinicians, laboratory scientists and pharma representatives. The widespread networking and exponential growth of post-conference interactions on social media have incentivised international scientific and medical experts to prioritise further collaborative study into topics such as mental health and patient-empowerment strategies; these topics will also serve as objectives for the next International Alport Workshop, which will be in 2023, respecting the established bi-annual cadence. In summary, there were no treatments in 2013 for Alport Syndrome, research was limited, and no pharmaceutical companies involved. 8 years on, having delivered five very successful international ‘lock-in’ workshops, there is now a vibrant international research community evidenced by the more than 250 researchers, with 50 new research projects featured at “The 2021 Online Workshop” with more than 15 pharmaceutical or biotech companies collaborating with the community and developing potential drug and gene therapy treatments with three being tested in international clinical trials.

**Fig. 5:**
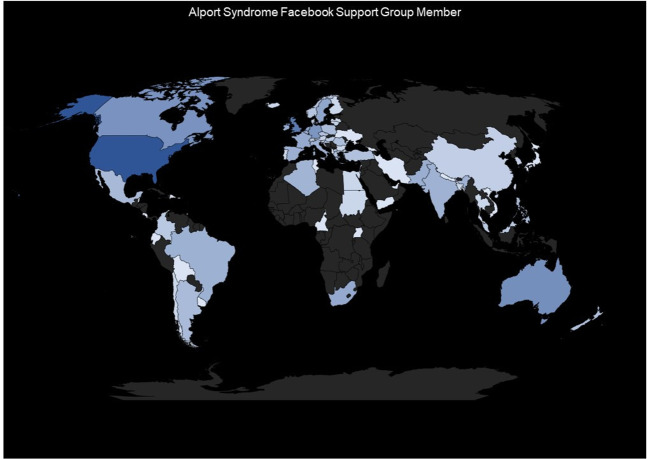
World map representation of AS patients distribution. World map represents the scale and scope of the ASF Facebook user community on a global scale, quantifying patients in a unique database.

The global participation of the AS patients around the world is represented in Fig. 5.
