# Correction: The 2019 and 2021 International workshops on Alport syndrome

**DOI:** 10.1038/s41431-023-01286-z

**Published:** 2023-01-24

**Authors:** Sergio Daga, Jie Ding, Constantinos Deltas, Judy Savige, Beata S. Lipska-Ziętkiewicz, Julia Hoefele, Frances Flinter, Daniel P. Gale, Marina Aksenova, Hirofumi Kai, Laura Perin, Moumita Barua, Roser Torra, Jeff H. Miner, Laura Massella, Danica Galešić Ljubanović, Rachel Lennon, Andrè B. Weinstock, Bertrand Knebelmann, Agne Cerkauskaite, Susie Gear, Oliver Gross, A. Neil Turner, Margherita Baldassarri, Anna Maria Pinto, Alessandra Renieri

**Affiliations:** 1 Medical Genetics, University of Siena, Siena, Italy; 2 Med Biotech Hub and Competence Center, Department of Medical Biotechnologies, University of Siena, Siena, Italy; 3 Peking University First Hospital, Beijing, China; 4 Biobank.cy Center of Excellence in Biobanking and Biomedical Research and University of Cyprus Medical School, Nicosia, Cyprus; 5 Department of Medicine, Melbourne and Northern Health, The University of Melbourne, Parkville, VIC 3050 Australia; 6 Rare Diseases Centre, Clinical Genetics Unit, Department of Biology and Medical Genetics, Medical University of Gdańsk, Gdansk, Poland; 7 Institute of Human Genetics, Klinikum rechts der Isar, Technical University of Munich, School of Medicine, Munich, Germany; 8 Department of Clinical Genetics, Guys’ and St Thomas’ NHS Foundation Trust, London, UK; 9 Department of Renal Medicine, University College London, London, UK; 10 Rare Renal Disease Registry, UK Renal Registry, Bristol, UK; 11 Y. Veltischev Research and Clinical Institute for Pediatrics at the Pirogov Russian National Research Medical University, Taldomskaya Street, 2, Moscow, 125412 Russia; 12 Department of Molecular Medicine, Kumamoto University, Kumamoto, Japan; 13 GOFARR Laboratory for Organ Regenerative Research and Cell Therapeutics in Urology, Saban Research Institute, Division of Urology, Children’s Hospital Los Angeles, Los Angeles, CA USA; 14 Department of Urology, Keck School of Medicine, University of Southern California, Los Angeles, CA USA; 15 Toronto General Hospital, Toronto General Research Institute, University of Toronto, Toronto, ON Canada; 16 Inherited Kidney Diseases, Nephrology Department, Fundació Puigvert, IIB-Sant Pau, Medicine Department, Universitat Autónoma de Barcelona, Barcelona, Spain; 17 Division of Nephrology, Washington University School of Medicine, St. Louis, MO 63110 USA; 18 Division of Nephrology, Department of Pediatric Subspecialties, Bambino Gesù Children’s Hospital, IRCCS, Rome, Italy; 19 University of Zagreb School of Medicine, Department of Pathology and Department of Nephropathology and Electron Microscopy Dubrava University Hospital, Zagreb, Croatia; 20 Wellcome Centre for Cell-Matrix Research, Division of Cell-Matrix Biology and Regenerative Medicine, School of Biological Sciences, Faculty of Biology Medicine and Health, The University of Manchester, Manchester Academic Health Science Centre, Manchester, UK; 21 Alport Syndrome Foundation, Phoenix, AZ USA; 22 Nephrology Department, Reference Center for Inherited Kidney Diseases (MARHEA), APHP, Necker Hospital, Paris University, Paris, France; 23 Faculty of Medicine, Vilnius University, Vilnius, Lithuania; 24 Vilnius University Hospital Santaros Klinikos, Vilnius, Lithuania; 25 Alport UK, Tetbury, UK; 26 Department of Nephrology and Rheumatology, University Medicine Goettingen, Gottingen, Germany; 27 Centre for Inflammation, University of Edinburgh, Edinburgh, UK; 28 Genetica Medica, Azienda Ospedaliero-Universitaria Senese, Siena, Italy

**Keywords:** Genetics research, Genetics

Correction to: *European Journal of Human Genetics* 10.1038/s41431-022-01075-0, published online 09 March 2022

The following acknowledgement was missing from this article.

This research has been supported (not financially) by “European Reference Network for Rare Kidney Disease, ERKNet”. This ERN is partly co-funded by the European Union within the framework of the Third Health Programme “ERN- 2016 - Framework Partnership Agreement 2017-2021”

